# Influenza Vaccination Performance Measurement Among Acute Care Hospital-Based Health Care Personnel — United States, 2013–14 Influenza Season

**Published:** 2014-09-19

**Authors:** Megan C. Lindley, Carolyn B. Bridges, Raymond A. Strikas, Elizabeth J. Kalayil, LaDora O. Woods, Daniel Pollock, Dawn Sievert

**Affiliations:** 1 Immunization Services Division, National Center for Immunization and Respiratory Diseases, CDC; 2 Division of Healthcare Quality Promotion, National Center for Emerging and Zoonotic Infectious Diseases, CDC

Annual influenza vaccination is recommended for all health care personnel (HCP) (1). In August 2011, the Centers for Medicare and Medicaid Services (CMS) published a final rule requiring acute care hospitals that participate in its Hospital Inpatient Quality Reporting Program to report HCP influenza vaccination data through the National Healthcare Safety Network (NHSN) beginning January 1, 2013 (2). Data reported by 4,254 acute care hospitals, covering the period October 1, 2013, through March 31, 2014, were analyzed to collect estimates of the proportion of HCP vaccinated nationally and by state for three groups: 1) employees, 2) licensed independent practitioners (LIPs), and 3) adult students/trainees and volunteers. Overall in the United States, 81.8% of hospital-based HCP were reported vaccinated, with the highest proportion (86.1%) among employees and the lowest (61.9%) among LIPs. The proportion reported vaccinated varied widely by state, with ranges of 69.0%–97.6% for employees, 33.8%–93.6% for LIPs, and 50.3%–96.3% for adult students/trainees and volunteers. Public reporting of vaccination data has been shown to increase HCP influenza vaccination coverage (3). These new NHSN data provide a baseline for measuring changes in future hospital-based reporting of HCP influenza vaccination.

NHSN is a secure, voluntary, web-based surveillance system managed by CDC, which allows NHSN participants in health care facilities to track and analyze data on health care–associated infection and prevention practices to determine the incidence of adverse events by facility, identify trends, and undertake local quality improvement activities to reduce health care-associated infection and associated adverse events (4). Data in this analysis were reported by NHSN participants in 4,254 acute care hospitals in 50 states and the District of Columbia. Hospitals reported data for three mutually exclusive HCP groups, regardless of clinical responsibility or patient contact: employees, LIPs, and adult students/trainees and volunteers (Box). For each group, hospitals reported the number of HCP who physically worked in the facility for at least 1 day from October 1, 2013, through March 31, 2014.

For HCP working in the hospital during the reporting period, NHSN participants reported one of four vaccination statuses: vaccinated, medically contraindicated, declined vaccination, or unknown. Vaccinated HCP were subdivided into two separately reported groups: those vaccinated at the facility where they worked, and those who provided written attestation or documentation of receiving vaccination elsewhere (e.g., physician’s office, pharmacy, or other retail location). HCP were considered contraindicated for influenza vaccination if they ever had a severe hypersensitivity reaction (e.g., anaphylaxis) to eggs or another vaccine component or had Guillain-Barré syndrome within 6 weeks of a previous influenza vaccination. HCP refusing vaccination for any other reason were reported as declining, and HCP not meeting the definition for any other vaccination status were reported as unknown. Because influenza vaccine might be available as early as July in some years, CDC instructed NHSN participants to report all vaccinations received from October 1 or when vaccine became available at their facility, whichever was earlier, through March 31.

Data were aggregated for all hospitals within a state and analyzed to calculate proportions of HCP reported as vaccinated for each of the three HCP groups by state and nationally. The proportion vaccinated was expressed as a percentage calculated as the number of HCP vaccinated at their facility plus the number who provided documentation of vaccination elsewhere, divided by the total number of HCP in that group, and multiplied by 100. The measure denominator included HCP with unknown vaccination status because ability to track and report HCP vaccination status is an important component of this performance measure. Aggregating vaccination data across facilities mitigated the effect of wide ranges among states in the number of reporting hospitals and the number of HCP working in those hospitals.

Overall in the United States, 81.8% of hospital-based HCP included in NHSN data reported receiving influenza vaccination during the 2013–14 influenza season, ranging from 62.4% in New Jersey to 96.4% in Maryland (Table). The reported proportion of HCP vaccinated was highest among employees (86.1%) and lowest among LIPs (61.9%); among adult students/trainees and volunteers, 79.9% were reported vaccinated. Similar patterns were noted at the state level, although in 10 states, the proportion of adult students/trainees and volunteers vaccinated was equal to or higher than the proportion among employees. The proportion vaccinated was lowest among LIPs in all but three states. Among LIPs, 35.0% had unknown vaccination status compared with 5.5% of employees and 16.5% of adult students/trainees and volunteers.

BOX **Definitions of health care personnel groups for National Healthcare Safety Network reporting — United States, 2013–14 influenza season**

**Employees**

All persons directly employed by the health care facility (i.e., receiving a direct paycheck from facility).

**Licensed independent practitioners**

Physicians, advanced practice nurses, and physician assistants who are affiliated with the health care facility, but are not directly employed by it.

**Adult students/trainees and volunteers**

Medical, nursing, or other health professional students, interns, medical residents, or volunteers aged ≥18 years who are affiliated with the health care facility, but are not directly employed by it.

The proportion reported vaccinated varied widely by state for all HCP groups: 69.0%–97.6% for employees, 33.8%–93.6% for LIPs, and 50.3%–96.3% for adult students/trainees and volunteers. NHSN participants in 13 states reported that 90% or more of hospital-based HCP overall were vaccinated. Vaccination of ≥90% of employees was reported by participants in 17 states, of LIPs by participants in two states, and of adult students/trainees and volunteers by participants in 11 states. A higher proportion of NHSN participants in states in the Midwest reported ≥90% of employees and HCP were vaccinated compared with other U.S. Census regions; this difference was less pronounced for adult students/trainees and volunteers. Only participants in the District of Columbia and Maryland reported vaccination of ≥90% of HCP in all three groups.

## Discussion

For the 2013–14 influenza season, NHSN reports showed 81.8% of acute care hospital-based HCP received influenza vaccination. The reported proportion of HCP vaccinated was highest among employees and lowest among LIPs, and varied by state. Just over one quarter of states reached the *Healthy People 2020* (HP2020) target of 90% influenza vaccination among HCP (5) in their hospitals. NHSN performance measurement data also revealed challenges in tracking LIP vaccination status, indicating additional work is needed for hospitals to accurately track vaccination of all HCP.

The Advisory Committee on Immunization Practices defines HCP as “all paid and unpaid persons working in health care settings who have the potential for exposure to patients and/or to infectious materials” (1). All HCP, regardless of employment status, should receive annual influenza vaccination to protect themselves and patients. Data in this analysis indicate that tracking vaccination of LIPs, who are likely to have substantial patient contact, was challenging; this group had the lowest proportion reported vaccinated and the highest proportion of HCP with unknown vaccination status nationally. Independent practitioners are highly mobile, can work in multiple facilities, and might enter hospitals infrequently. Many LIPs likely receive influenza vaccination outside of reporting facilities; therefore, the actual proportion of LIPs vaccinated might be higher than reported. Improvements in hospitals’ ability to track LIPs likely will result in higher reported proportions vaccinated in future influenza seasons.

Data on approximately 8 million HCP were reported by 4,254 acute care hospitals, which represents 85% of community hospitals* in the United States (6). These data represent the most complete accounting available of hospital-based HCP influenza vaccination measurement. Compared with unpublished data from the 2012–13 influenza season, when hospitals first reported HCP summary influenza vaccination to NHSN, the proportion reported vaccinated is higher for all HCP groups, and nearly 800,000 more HCP were included in 2013–14 reporting.†

CDC uses multiple systems to monitor HCP influenza vaccination. The National Health Interview Survey (NHIS) is the data source for measuring progress toward the HP2020 target for HCP influenza vaccination. NHIS is an in-person survey that collects self-reported vaccination status; it covers all health care settings but does not directly target HCP, resulting in small HCP sample sizes. The HCP opt-in Internet panel survey, conducted for CDC since 2010, collects self-reported vaccination status among HCP who have volunteered to be contacted for online surveys (7,8). The Internet panel survey provides timelier and more detailed data than NHIS, including coverage estimates by occupation type and health care setting, but the use of nonprobability sampling limits the generalizability of results. Vaccination status reported through NHSN is likely more accurate than self-report because of documentation requirements for some data elements, and NHSN provides state-level estimates. However, NHSN does not currently cover nonhospital settings§ nor provide the same level of detail about vaccination by occupation as the Internet panel survey. Because NHSN includes HCP with unknown vaccination status in the measure denominator, NHSN data represent minimum estimates of vaccination coverage. When restricted to those with known vaccination status, NHSN estimates of proportion vaccinated were 92.2% for HCP overall, 91.2% for employees, 95.4% for LIPs, and 95.7% for adult students/trainees and volunteers. Estimates of HCP influenza vaccination differ among the three systems and are not directly comparable (7). Although each has strengths and weaknesses, taken together, CDC’s monitoring systems provide a comprehensive picture of U.S. HCP influenza vaccination.

The findings in this report are subject to at least two limitations. First, data reported by hospitals to NHSN are not validated by CDC. However, a validation study conducted prior to NHSN reporting indicated hospital-reported HCP vaccination data were catgorized in a manner consistent with measure definitions (9). Second, employment practices vary by state and hospital; therefore, NHSN-defined HCP categories might not represent the same mix of job functions and personnel across facilities. For example, some hospitals directly employ the majority of their physicians and nurses, whereas others rely on individual contracts or staffing agencies to supply these personnel.

Public reporting of HCP vaccination data is an important strategy to increase influenza vaccination coverage. A voluntary public reporting program among Iowa hospitals resulted in an increase of 20 percentage points in median employee influenza vaccination coverage over 4 years (3). The *Guide to Community Preventive Services* recommends assessment and feedback on vaccination rates as an evidence-based approach to increase vaccination coverage (10). Facility-level reports of HCP influenza vaccination will be published by CMS on its Hospital Compare website¶ in 2014. The CMS Hospital Inpatient Quality Reporting program comprises a list of performance measures, including HCP influenza vaccination, which acute care hospitals must report annually to CMS. Hospitals failing to report all required measures can be subject to a decrease in their annual payment update from CMS. This provides a financial incentive for acute care hospitals to report HCP influenza vaccination data to NHSN, contributing to completeness of reporting. Data in this report provide a baseline for measuring changes in hospital-based HCP vaccination reporting in future influenza seasons. States and hospitals can use these data to evaluate the effectiveness of efforts to increase HCP influenza vaccination in pursuit of the HP2020 target of 90% vaccination.

What is already known on this topic?

The Advisory Committee on Immunization Practices recommends annual influenza vaccination for all health care personnel (HCP) to reduce influenza-related morbidity and mortality in health care settings.

What is added by this report?

Nationally, 81.8% of HCP included in National Healthcare Safety Network data were reported as receiving influenza vaccination during the 2013–14 influenza season. Reported proportion of HCP vaccinated was highest among employees (86.1%) and lowest among licensed independent practitioners (61.9%) and varied widely by state for all HCP groups.

What are the implications for public health practice?

Public reporting of vaccination data has been shown to increase HCP influenza vaccination coverage. These data provide a baseline from which to measure changes in reported hospital-based HCP vaccination and in ability to track HCP vaccination. Improvements in hospitals’ ability to track licensed independent practitioners might result in higher reported vaccination among these HCP in future influenza seasons.

## Figures and Tables

**TABLE:** Proportion of health care personnel (HCP) reported vaccinated for influenza by reporting acute care hospitals, by personnel group and state — National Healthcare Safety Network, United States, 2013–14 influenza season

State	% of employees	% of licensed independent practitioners	% of adult students/trainees and volunteers	% of all HCP
Alabama	76.5	40.8	78.6	72.2
Alaska	78.7	55.9	53.1	72.8
Arizona	82.0	62.7	70.6	76.8
Arkansas	88.0	44.3	78.1	82.4
California	83.4	62.3	79.1	79.3
Colorado	97.1	88.6	95.0	94.9
Connecticut	87.6	72.5	87.6	85.6
Delaware	93.7	71.7	95.6	92.1
District of Columbia	96.4	92.0	93.6	95.5
Florida	72.4	33.8	67.0	64.4
Georgia	88.7	69.5	89.4	86.2
Hawaii	69.0	57.6	62.3	66.6
Idaho	86.9	34.5	74.5	78.8
Illinois	87.8	62.8	81.6	83.3
Indiana	90.3	73.4	86.4	87.5
Iowa	95.2	77.6	83.0	91.2
Kansas	92.0	69.6	92.2	88.7
Kentucky	81.5	45.8	68.3	75.2
Louisiana	77.0	39.6	77.1	71.2
Maine	86.5	70.1	86.8	85.8
Maryland	97.3	93.6	95.2	96.4
Massachusetts	87.1	78.0	87.3	86.1
Michigan	87.0	49.3	78.6	81.5
Minnesota	79.4	61.0	61.9	75.3
Mississippi	75.6	50.6	75.2	73.6
Missouri	93.0	64.9	85.3	88.4
Montana	87.4	62.0	75.0	83.8
Nebraska	94.3	73.4	94.3	91.5
Nevada	88.2	44.6	80.6	75.8
New Hampshire	94.5	82.2	93.4	93.1
New Jersey	70.8	39.1	50.3	62.4
New Mexico	83.1	41.5	74.1	76.4
New York	85.9	66.3	84.2	83.3
North Carolina	94.8	82.0	88.4	92.4
North Dakota	91.3	75.5	90.7	90.2
Ohio	87.1	58.3	72.0	82.0
Oklahoma	87.1	64.1	81.5	83.6
Oregon	82.3	58.1	63.0	76.7
Pennsylvania	86.2	62.8	82.5	82.1
Rhode Island	89.7	87.7	96.3	90.4
South Carolina	85.0	67.8	80.2	82.1
South Dakota	94.9	78.8	93.3	93.5
Tennessee	87.2	57.9	79.1	81.9
Texas	91.3	59.3	80.3	83.4
Utah	97.6	79.8	92.9	94.0
Vermont	78.3	46.5	62.0	74.7
Virginia	88.6	71.3	86.4	85.5
Washington	90.9	70.1	89.6	87.6
West Virginia	82.4	47.1	71.8	77.6
Wisconsin	92.2	88.3	86.6	90.7
Wyoming	89.3	71.7	62.8	82.5
**U.S. overall**	**86.1**	**61.9**	**79.9**	**81.8**
No. of HCP*	5,683,406	1,154,376	1,168,861	8,006,643
*Range*	*69.0–97.6*	*33.8–93.6*	*50.3–96.3*	*62.4–96.4*

* HCP are reported by each facility in which they worked. Therefore, persons might be counted more than once, and the actual number of unique HCP likely is lower.

## Footnotes

* The American Hospital Association defines community hospitals as all nonfederal, short-term general, and other special hospitals (including obstetrics and gynecology; eye, ear, nose, and throat; rehabilitation; orthopedic; and other individually described specialty services) accessible by the general public.

† Data for the 2012–13 season will not be published by CDC or CMS because reporting was required beginning January 1, 2013; therefore, reported data for 2012–13 might not cover the entire influenza season.

§ Estimates of influenza vaccination coverage among health care personnel working in settings other than acute care hospitals can be obtained for selected states via the optional industry and occupation module of the Behavioral Risk Factor Surveillance System (BRFSS). This module was implemented in the 2013 BRFSS survey; module questions are available at http://www.cdc.gov/brfss/questionnaires/pdf-ques/2013%20brfss_english.pdf.

¶ Available at http://www.medicare.gov/hospitalcompare/search.html.
