# Erratum

**Published:** 2014-09-26

## Vol. 63, No. 37

In the report, “Influenza Vaccination Performance Measurement Among Acute Care Hospital-Based Health Care Personnel — United States, 2013–14 Influenza Season,” on page 814, in the first full paragraph of the second column, the third sentence should read, “However, a validation study conducted prior to NHSN reporting indicated hospital-reported HCP vaccination data were **categorized** in a manner consistent with measure definitions (*9*).”

